# The small molecule peptide ANXA114‐26 inhibits ovarian cancer cell proliferation and reverses cisplatin resistance by binding to the formyl peptide receptors receptor

**DOI:** 10.1002/ccs3.12058

**Published:** 2024-12-19

**Authors:** Nana Li, Peihua Yan, Ling Guo, Huiyan Wang, Baohong Cui, Lichen Teng, Yajuan Su

**Affiliations:** ^1^ Department of Clinical Laboratory Harbin Medical University Cancer Hospital Harbin China

**Keywords:** ANXA114‐26, cisplatin resistance, cyclin D1, FPR, NF‐ĸBp65, ovarian cancer

## Abstract

Chemo‐resistance in ovarian cancer is currently a major obstacle to the treatment and recovery of ovarian cancer. Therefore, identifying factors associated with chemo‐resistance in ovarian cancer may reverse chemo‐sensitization. Using isobaric tags for relative and absolute quantitation (ITRAQ) technology, we found a small molecule peptide with annexin 1 (ANXA1) as a precursor protein. Then, we explored the effects and mechanisms of this small molecule peptide on the proliferation, apoptosis, and drug resistance of ovarian cancer resistant cells through CCK‐8, EdU cell proliferation assay, Annexin V‐FITC/PI assay, Western blot,qRT‐PCR. ANXA114‐26 was highly expressed in the serums of sensitive patients. ANXA114‐26 promoted apoptosis of ovarian cancer cells and increased the sensitization of ovarian cancer cells to cisplatin. The ANXA114‐26 and ANXA1 competitively bind formyl peptide receptors (FPR). ANXA114‐26 decreased multidrug resistance‐associated protein 1 (MRP1) expression in ovarian cancer cells through the FPR/Cyclin D1/NF‐ĸBp65 pathway. We found a peptide derived named ANXA114‐26 in the serum of ovarian cancer patients. It can reduce ovarian cancer cell proliferation and reduce MRP1 expression through the FPR/Cyclin D1/NF‐ĸBp65 pathway.

## Graphical abstract

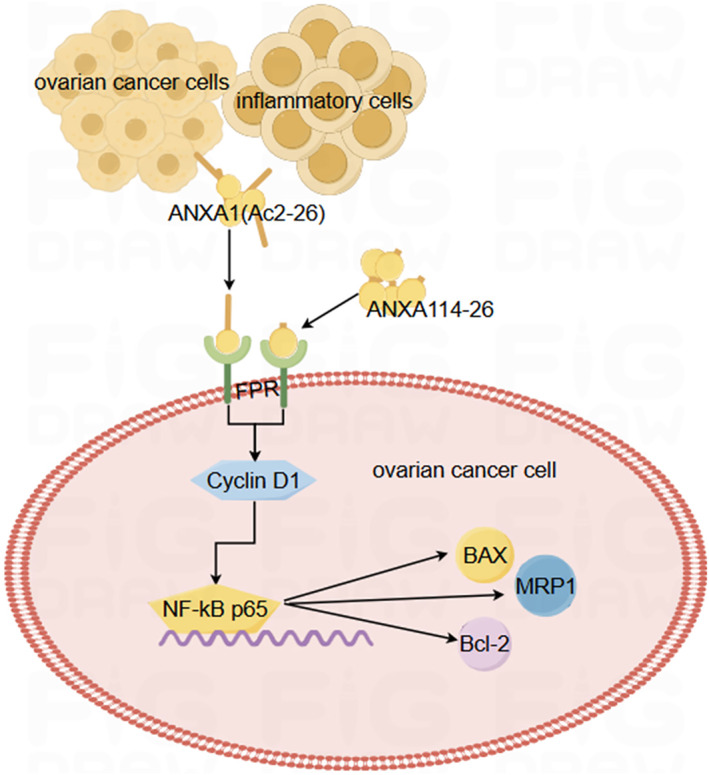

## 1 INTRODUCTION

Ovarian cancer is a common malignant tumor that highly threatens women’s health. Its 5‐year survival rate is 50%, which is lower than the 5‐year survival rate of total cancer.^1^ Currently, the treatment for ovarian cancer is aggressive primary surgery followed by cisplatin‐based chemotherapy. However, because of the high rate of recurrence and progressive disease resistant to cisplatin, prognosis of ovarian cancer is very poor.

Organic compounds consisting of three or more amino acid molecules linked by peptide bonds are called polypeptides. Polypeptides are found in small amounts in the human body, but they are widely distributed and participate in various physiological and pathological activities.^2^, ^3^ Presently, researchers have found many mimetic peptides involved in tumor drug resistance, which are partially identical to their precursor proteins in amino acid sequences or molecular structures. These polypeptides can competitively bind to ligand proteins with their precursor proteins, blocking the drug‐resistant effects of the ligand proteins.^4^, ^5^ Some of them can mimic the biological function of precursor proteins, thereby restoring or increasing the sensitivity of tumor cells to chemotherapy agents.^6^, ^7^

The biological role of ANXA1 involves multiple physiological and pathological processes.^8^, ^9^, ^10^ ANXA1 is also involved in the process of tumor resistance, but whether it alleviates drug resistance or promotes drug resistance is still controversial.^11^, ^12^, ^13^ ANXA1 and its N‐telopeptides function outside the cell through the receptor FPR on the cell membrane.^10^, ^14^ It has been found that FPR involves the occurrence, drug resistance, and staging of tumor .^10^, ^15^, ^16^, ^17^ Hypoxia‐induced increase in FPR expression decreases the sensitivity of ovarian cancer cells to cisplatin^18^; The expression of FPR1 in bladder cancer drug‐resistant cells was higher than that in parental cells, and the activation of FPR1 promoted the expression of MRP1 mRNA.^19^

Serum from cisplatin‐resistant/sensitive patients with ovarian cancer was collected and peptide analysis was performed using iTRAQ to screen out a peptide ANXA114‐26 associated with drug resistance. We propose the hypothesis that ANXA114‐26 inhibits ovarian cancer cell proliferation and reverses cisplatin resistance by binding to FPR receptors. This study will explore its relationship with drug resistance in ovarian cancer cells and its specific mechanism at the cytological level.

## 2 MATERIALS AND METHODS

### 2.1 Reagents

Cisplatin, Ac2‐26 and Boc‐MLF were purchased from Glpbio (Glpbio, Inc., California, USA). β‐actin,Bax,Bcl‐2,MRP1, Cyclin D1, ANXA1 and NF‐ĸBp65 antibodies were purchased from Wanleibio (Wanleibio, China). Goat anti‐rabbit secondary antibodies were purchased from Beyotime (Beyotime, Shanghai, China). Small molecule peptides ANXA114‐26 sequence (IENEEQEYVQTVK) is synthesized by Bankpeptide Biological Technology Co (Bankpeptide, China).

### 2.2 Cell culture

SKOV3 cells were cultured in McCoy’s 5A medium (GenomBio, China) containing 10% fetal bovine serum (ExCellBio, China) and Penicillin‐Streptomycin Solution (Seven, China). SKOV3/DDP cells were cultured in McCoy’s 5A medium (GenomBio, China) containing 10% fetal bovine serum (ExCellBio, China), Penicillin‐Streptomycin Solution (Seven, China) and 500 ng/mL Cisplatin (DDP) (Solarbio, China).

### 2.3 Cell Counting Kit‐8

The cell viability was estimated by Cell Counting Kit‐8 (CCK‐8 assay) (Seven, China) according to the manufacturer’s protocol. Briefly, 3 × 103 cells grown in 96‐well plates were incubated with CCK‐8 solution for 1.5 h at 37°C, after which the absorbance of each well at 450 nm was recorded. The viability of SKOV3 and SKOV3/DDP cells was calculated as follows: (Sample‐blank)/(control‐blank) × 100.

### 2.4 EdU cell proliferation assay

First, 3 × 103 cells grown in 96‐well plates were incubated with EdU solution for 2 h at 37°C, then it went through the process of fixation, washing, and permeability. The samples were then incubated with the click reaction solution for 30 min at room temperature in the dark. The nuclei were stained with Hoechst. Finally, fluorescence detection is performed.

### 2.5 Apoptosis detection

Annexin V‐FITC/PI kit (Wanlei Biotechnology, China) was used to detect apoptosis. Each group of cells was digested with trypsin, and then centrifuged at 1500 r/min. Resuspend the cells in 500 μl binding buffer, add 10 μl PI and 5 μl Annexin‐FITC, incubate for 15 min in the dark. Then cells were detected by a flow cytometer.

### 2.6 Western blot analysis

First, the proteins were extracted with a RIPA lysis buffer from cells, and the concentration was determined via a bicinchoninic acid (BCA) assay. Each sample was electrophoresed on an SDS‐polyacrylamide gel and transferred to PVDF membranes. The PVDF membranes were blocked with PBS containing 5% skim milk and incubated with primary antibodies (anti‐Bax, 1:1000; anti‐Bcl‐2, 1:1000; anti‐MRP1, 1:1000; anti‐Cyclin D1, 1:1000; anti‐ANXA1, 1:1000; anti‐NF‐ĸBp65, 1:1000; anti‐β‐actin, 1:1000) at 4°C overnight. Next, the membranes were washed with TBST and then incubated with secondary antibodies for 1 h at room temperature. Finally, the PVDF membranes were visualized by a Tanon 5200 series fully automated chemiluminescence image analysis system.

### 2.7 Quantitative reverse transcription polymerase chain reaction (qRT‐PCR)

First, total RNA was extracted by TRIzol reagent (Seven, China). Then, cDNA was synthesized from the RNA with *Evo M‐MLV* RT Reaction Mix (Accurate Biology, China) and use SYBR Green *Pro Taq* HS Premix for quantitative PCR (Accurate Biology, China). Human primers sequences are as follows: GAPDH(F′): ;5′‐ GAGTCAACGGATTTGGTCGT ‐3′, GAPDH (R′): 5′‐ TTGATTTTGGAGGGATCTCG‐3′; ANXA1(F′): 5′‐GAGGAGGTTGTTTTAGCTCTGC‐3′, ANXA1 (R′): 5′‐ AGCAAAGCGTTCCGAAAATCT‐3′; BAX (F′): 5′‐ TGGCAGCTGACATGTTTTCTGAC‐3′, BAX (R′): 5′‐ TCACCCAACCACCCTGGTCTT‐3′; Bcl‐2(F′): 5′‐AAGACTGCTTGGCCCATCTT‐3′,Bcl‐2 (R′): 5′‐ CGCTCAAAGGTTCTTGACCA‐3′; MRP1(F′): 5′‐GTCGGACCACCATTGTGATAG‐3′, MRP1 (R′): 5′‐ CATTTCCTGCTGTCTGCATTGTG‐3′; NF‐ĸBp65(F′): 5′‐AATCCAGTGTGTGAAGAAGC‐3′, NF‐ĸBp65(R′): 5′‐ GCTGCTCTTCTATAGGAACT‐3′; Cyclin D1(F′): 5′‐ GAGGCGGAGGAGAACAAACA‐3′, Cyclin D1 (R′): 5′‐ GGAGGGCGGATTGGAAATGA ‐3′.

### 2.8 Statistical analysis

The data results in this study are at least three replicates, expressed as mean ± SD. The data was analyzed by GraphPad Prism8. Student’s *t* test was used to compare the differences between two groups. One‐way analysis of variance (ANOVA), was performed for comparisons of the differences among more than two groups. *p* values < 0.05 were considered statistically significant.

## 3 RESULTS

### 3.1 Screening of ANXA114‐26 in cisplatin‐resistant/sensitive serum for ovarian cancer

Initially, we screened differential expression of small molecule peptides with iTRAQ technology in the serum of cisplatin‐resistant/sensitive patients with ovarian cancer, and the results showed that a total of 41 differentially expressed small molecule peptides were identified (fold change >2, *p* < 0.05) (Table 1).

**TABLE 1:** Relative expression of target peptides in cisplatin‐resistant/sensitive serum of ovarian cancer.

Amino acid sequence	Precursor proteins	R/S *p*‐value	R/S diffstat
AEPAVQRTLLEK	CD99	0.027908344	Down
SDKPDMAEIEKFDKSK	Tβ4	0.001450893	Down
GSGGGSFGDNLVTR	LMNA	0.029291407	Down
GGGSFGDNLVTR	LMNA	0.012609003	Down
STPLGQQQPAPR	LTBP2	0.043236733	Down
IENEEQEYVQTVK	ANXA1	0.040350765	Down
SGGGSFGDNLVTR	LMNA	0.013669379	Down
GGSFGDNLVTR	LMNA	0.044314649	Down
RGAPAAATAPAPTAHKA	H1X	0.033926143	Down
TTAAAVASTGPSSR	MAP4	0.029422526	Down
VELEDWAGNEAYAEYHFR	FIBA	0.011367614	Down
AGFAGDDAPR	C3	0.04797934	Down
ADSGEGDFLAEGGGVRG	FIBA	0.048735954	Down
GDAGAPGAPGGKGDAGAPGERGPPG	COL3A1	0.035792472	Down
DGEAGAQGPPGP	COL1A1	0.048672979	Down
TTTLSGTAPAAGVVPSR	MAP4	0.048518656	Up
LDSLPLVDTHSK	VIME	0.049119088	Up
SETAPAAPAAPAPAEKTPVK	H14	0.010023351	Up
ASSDIQVKELEK	STMN1	0.016892779	Up
SETAPAAPAAPAPAEKTPVKK	H14	0.04410468	Up
SDAAVDTSSEITTKDLK	PTMA	0.021917774	Up
ATDTSQGELVHPK	HP1B3	0.003744965	Up
VPDLVPGNFK	FIBA	0.04573196	Up
VHLTPEEK	HBB	0.02682247	Up
KPVPDLVPGNFK	FIBA	0.049215437	Up
SPADKTNVK	HBA	0.000947803	Up
QLQKVPPEWK	FIBA	0.022897187	Up
AIQLTYNPDESSKPN	FIBA	0.020660376	Up
VLSPADKTNVK	HBA	0.011778286	Up
ETIEQEK	Tβ4	0.047943912	Up
NALLSLAK	ANXA1	0.029729648	Up
DVRWPTETDVSSAK	MAP4	0.017925689	Up
TVIGPDGHK	FIBA	0.004127417	Up
GSESGIFTNTK	FIBA	0.02422271	Up
QFTSSTSYNR	FIBA	0.037250804	Up
GDSTFESK	FIBA	0.048941087	Up
SEETKENEGFTVTAEGK	C3	0.039574595	Up
ADSGEGDFLAEGGGVR	FIBA	0.035897917	Up
EDPQGDAAQKTDTSHHDQDHPTF	SERPINA1	0.023789753	Up
GLLGSAQ	BRICD5	0.049738798	Up
DSGEGDFLAEGGGVR	FIBA	0.024975345	Up

We supposed that chemo‐resistance would be reversed by increasing the concentration of small molecule peptides, which were down‐regulated in the serums of patients with chemo‐resistance. After searching databases and related literature, we came to our attention with a small peptide sequence of IENEEQEYVQTVK, which was derived from amino acids 14–26 of ANXA1, so it was named ANXA114‐26. While the role of related peptides with ANXA1 as a precursor protein in tumors has been extensively documented,^9^, ^20^ to the best of our knowledge, the small molecule peptide ANXA114‐26 has never been reported.

### 3.2 ANXA114‐26 inhibits ovarian cancer cell proliferation and reduces drug‐resistant protein expression

**FIGURE 1:**
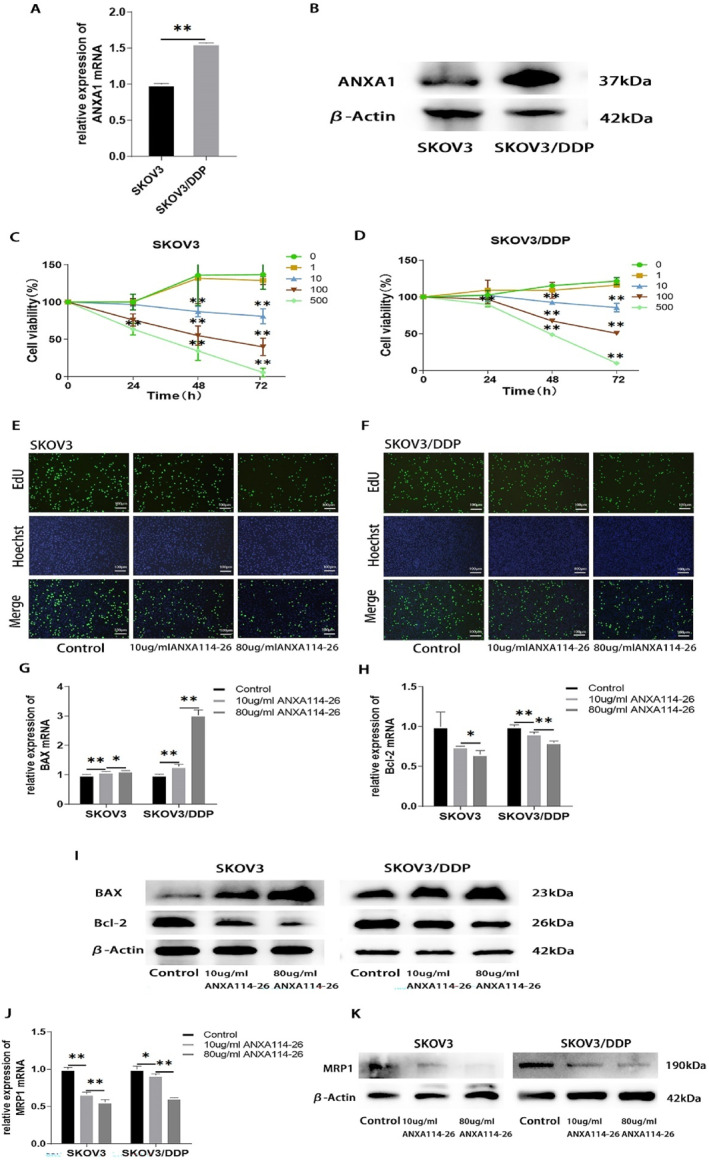
ANXA114‐26 inhibits ovarian cancer cell proliferation and reduces drug‐resistant protein expression. (A) Differences in mRNA expression of ANXA1 between SKOV3 and SKOV3/DDP cell lines. (B) Differences in protein expression of ANXA1 between SKOV3 and SKOV3/DDP cell lines. (C) Changes in cell viability in SKOV3 cell lines. (D) Changes in cell viability in SKOV3/DDP cell lines. (E) The cell proliferation rate of SKOV3 cell line was detected by EdU. (F) The cell proliferation rate of SKOV3/DDP cell line was detected by EdU. (G) The relative expression of BAX mRNA. (H) The relative expression of Bcl‐2 mRNA. (I) Protein expression levels of BAX and Bcl‐2. (J) The relative expression of MRP1 mRNA. (K) Protein expression levels of MRP1. The data are represented as the mean ± SD. The data were independently replicated in triplicate., *p < 0.05, **p < 0.01.

To identify relationship between expression of ANXA1 and chemo‐resistance in ovarian cancer, we examined the difference in the expression of ANXA1 in SKOV3 and SKOV3/DDP cell lines, and the PCR and Western blotting results showed that the expression of ANXA1 in SKOV3/DDP was increased (Figure 1A–B). The results indicated that the expression level of ANXA1 was closely related with chemo‐resistance in ovarian cancer cells, which were low expression in parental cells and high expression in drug‐resistant cells. We hypothesize that ANXA114‐26 and ANXA1 may competitively bind to the same ligand FPR, and ANXA1 cannot bind to FPR when ANXA114‐26 binds to the FPR ligand. Therefore, ANXA1‐mediated cisplatin resistance is reversed in ovarian cancer cells.

To explore the effect of ANXA114‐26 on the proliferative activity of ovarian cancer cells. We incubated the ovarian cancer parental cell line SKOV3 and the drug‐resistant cell line SKOV3/DDP with different concentrations of ANXA114‐26. Cell viability is assessed by CCK‐8 assay after 24, 48, and 72 h, respectively. The results showed that after 24 h of incubation, the cell proliferation of the 500 μg/mL ANXA114‐26 group significantly decreased compared to the control group; after 48 and 72 h of incubation, compared with the control group, the cell proliferation of ANXA114‐26 groups at 10 μg/mL, 100 μg/mL, and 500 μg/mL significantly decreased, and the decrease in cell proliferation gradually increased with increasing incubation concentration and time. (Figure 1C–D). Given the toxicity and effects of cisplatin, 10 μg/mL and 80 μg/mL ANXA114‐26 were used in the following experimental concentrations.

SKOV3 and SKOV3/DDP cells were incubated ANXA114‐26 for 48 h, and the proliferation of SKOV3 and SKOV3/DDP cells was detected by EdU assay. The results showed that ANXA114‐26 decreased the proliferation rate of SKOV3 and SKOV3/DDP cells in the concentration‐dependent manner (Figure 1E–F).

Next, SKOV3 and SKOV3/DDP cells were incubated ANXA114‐26 for 48 h, and the mRNA and protein expressions of pro‐apoptotic protein BAX and anti‐apoptotic protein Bcl‐2 were detected by qRT‐PCR and Western blot. The results showed that ANXA114‐26 could upregulate the expression of BAX and downregulate the expression of Bcl‐2 in both cell lines (Figure 1G–I). Moreover, ANXA114‐26 could downregulate mRNA and protein expression of MRP1 in SKOV3 and SKOV3/DDP cell lines (Figure 1J–K). In summary, ANXA114‐26 can inhibit the proliferation of ovarian cancer cells and reverse the resistance to cisplatin.

### 3.3 The combination of ANXA114‐26 with cisplatin inhibited the proliferation of ovarian cancer cells and reduced the expression of cisplatin‐resistant proteins

**FIGURE 2:**
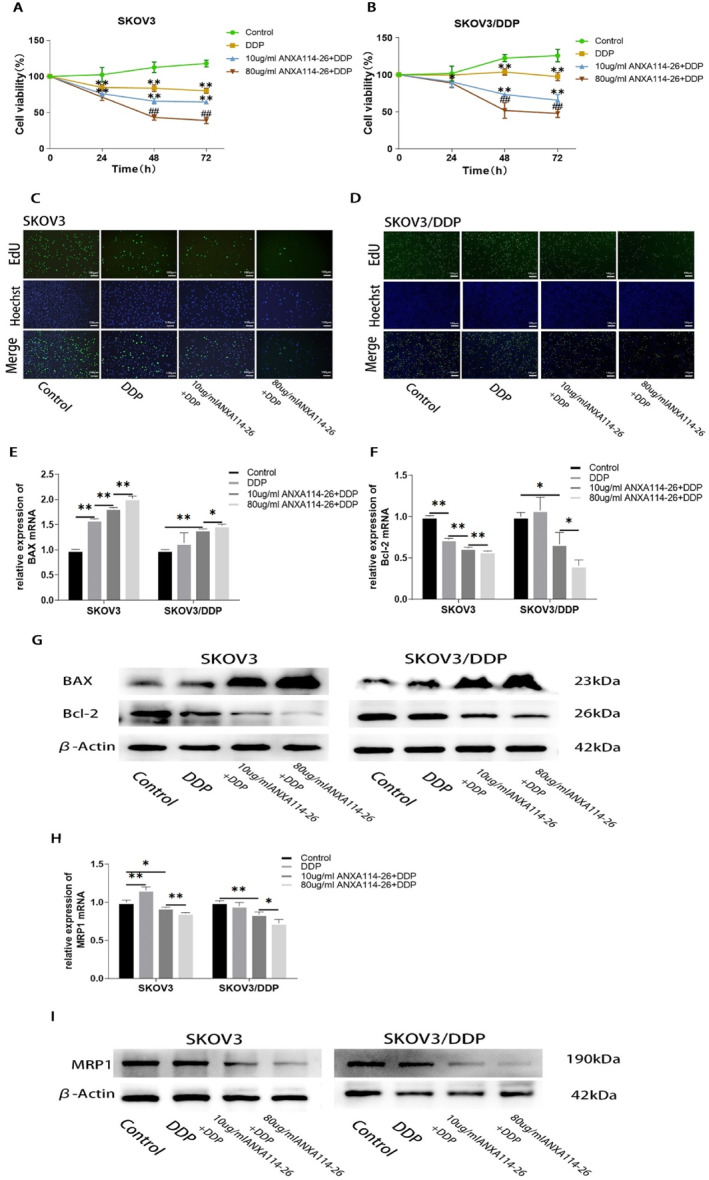
The combination of ANXA114‐26 and cisplatin inhibits the proliferation of ovarian cancer cells and reduces the expression of drug‐resistant proteins. (A) Changes in cell viability in SKOV3 cell lines. (B) Changes in cell viability in SKOV3/DDP cell lines. (C) The cell proliferation rate of SKOV3 cell line was detected by EdU. (D) The cell proliferation rate of SKOV3/DDP cell line was detected by EdU. (E) The relative expression of BAX mRNA. (F) The relative expression of Bcl‐2 mRNA. (G) Protein expression levels of BAX and Bcl‐2. (H) The relative expression of MRP1 mRNA. (I) Protein expression levels of MRP1. The data are represented as the mean ± SD. The data were independently replicated in triplicate., *p < 0.05, **p < 0.01. Compared to 10 μg/mlANXA114‐26+ DDP, ^#^ *p* < 0.05, ^##^ *p* < 0.01.

We applied ANXA114‐26 in combination with cisplatin to observe the effect on ovarian cancer cell proliferation. The results of CCK‐8 and EdU showed that compared with the control group, the cisplatin treatment group significantly reduced the proliferation of SKOV3 cells, but had no significant inhibition on the proliferation of SKOV3/DDP cells. In the ANXA114‐26 combined with cisplatin treatment group, the proliferation of both cells decreased significantly in the concentration of ANXA114‐26 dependent manner (Figure 2A–D). Compared with the control group, the expression of Bcl‐2 were significantly reduced in SKOV3 cells with cisplatin alone, but the expression of Bcl‐2 in SKOV3/DDP cells was not reduced, and the expression of Bcl‐2 in both cells was significantly decreased with the increase of ANXA114‐26 concentration in the group of combination of ANXA114‐26 with cisplatin. Compared with the control group, the expression of BAX in SKOV3 and SKOV3/DDP cells was slightly increased in the cisplatin treatment alone, and significantly increased in both cells with the increase of ANXA114‐26 concentration in the ANXA114‐26 combined cisplatin treatment group (Figure 2E–G). The above results showed that cisplatin alone could inhibit the proliferation of SKOV3 cells, but the proliferation of SKOV3/DDP cells was not inhibited or slightly inhibited, however, the combination of cisplatin with ANXA114‐26 significantly inhibited the proliferation of both types of cells.

Subsequently, the results showed that the expression of MRP1 has no significant difference in the SKOV3 and SKOV3/DDP cells in the cisplatin‐treated group, compared with the control cells. In the ANXA114‐26 combined with cisplatin treatment group, the expression of MRP1 decreased with the increase of ANXA114‐26 concentration (Figure 2H–I). The above results indicated that the combination of ANXA114‐26 and cisplatin could reduce the expression of MRP1 in SKOV3 and SKOV3/DDP cells, and reverse the chemo‐resistance of ovarian cancer cells.

### 3.4 ANXA114‐26 promoted apoptosis and reduces drug resistance in ovarian cancer cells by competitively binding to the FPR receptor with ANXA1

**FIGURE 3:**
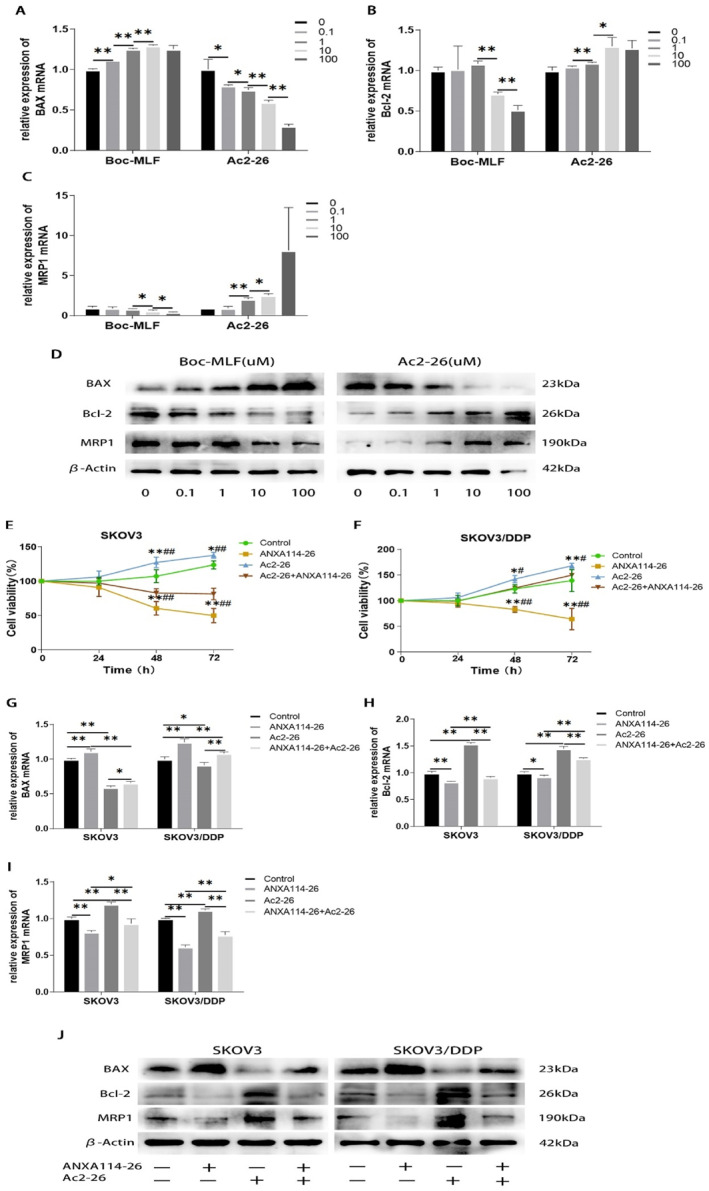
ANXA114‐26 promotes apoptosis and reduces drug resistance in ovarian cancer cells by competitively binding to the FPR receptor with ANXA1. (A) The relative expression of BAX mRNA. (B) The relative expression of Bcl‐2 mRNA. (C) The relative expression of MRP1 mRNA. (D) Protein expression levels of BAX,Bcl‐2 and MRP1. (E) Changes in cell viability in SKOV3 cell lines. (F) Changes in cell viability in SKOV3/DDP cell lines. (G) The relative expression of BAX mRNA. (H) The relative expression of Bcl‐2 mRNA. (I) The relative expression of MRP1 mRNA. (J) Protein expression levels of BAX,Bcl‐2 and MRP1. The data are represented as the mean ± SD. The data were independently replicated in triplicate., *p < 0.05, **p < 0.01. Compared to ANXA114‐26 + Ac2‐26, ^#^ *p* < 0.05, ^##^ *p* < 0.01.

Ac2‐26 is a peptide derived from ANXA1 N‐terminal positions 2–26, which mimics the biological effects of ANXA1.^9^, ^20^ ANXA1 is secreted outside the cell and exerts its biological function through the receptor FPR (including FPR1, FPR2, and FPR3) on the cell membrane.^8^, ^10^, ^14^ Boc‐MLF is a commonly used FPR1 inhibitor.^21^ Taking SKOV3/DDP cells as an example, we investigated the effects of Boc‐MLF and Ac2‐26 on the proliferation and drug resistance of ovarian cancer cells. The mRNA and protein expressions of BAX, Bcl‐2 and MRP1 were detected by qRT‐PCR and Western blot. The results showed that the expression of BAX in Boc‐MLF treated cells increased, and the expression levels of Bcl‐2 and MRP1 decreased. The expression of BAX in Ac2‐26 treated cells decreased, and the expression of Bcl‐2 and MRP1 increased (Figure 3A–D). The above results indicated that the inhibition of FPR1 by Boc‐MLF would lead to an increase in apoptosis and a decrease in MPR1 expression. Ac2‐26 can activate FPR and promote ovarian cancer cell proliferation and MRP1 expression, which is consistent with the high expression of ANXA1 in SKOV3/DDP that we detected.

In order to further explore whether ANXA114‐26 regulates ovarian cancer cells through FPR receptors, we used CCK‐8 assay to detect whether ANXA114‐26 can reverse the proliferative effect of Ac2‐26. The results showed that the Ac2‐26 group promoted cell proliferation and ANXA114‐26 inhibited cell proliferation compared to the control group. Compared with the Ac2‐26 group, the cell viability of the ANXA114‐26 + Ac2‐26 group decreased (Figure 3E–F), indicating that ANXA114‐26 could inhibit the proliferative effect of Ac2‐26. The mRNA and protein expressions of BAX, Bcl‐2 and MRP1 were detected by qRT‐PCR and Western blot, and the results showed that compared with the ANXA114‐26 group and the Ac2‐26 group, the expression levels of BAX in the ANXA114‐26 + Ac2‐26 group were higher than those in the Ac2‐26 group and lower than those in the ANXA114‐26 group. The expression levels of Bcl‐2 and MRP1 were lower than those in the Ac2‐26 group and higher than those in the ANXA114‐26 group (Figure 3G–J). These results suggest that ANXA114‐26 reverses the effect of Ac2‐26 on ovarian cancer cell proliferation and drug resistance through binding to FPR.

### 3.5 ANXA114‐26 may affect the proliferation and drug resistance of ovarian cancer cells through the Cyclin D1 and NF‐ĸB signaling pathways

**FIGURE 4:**
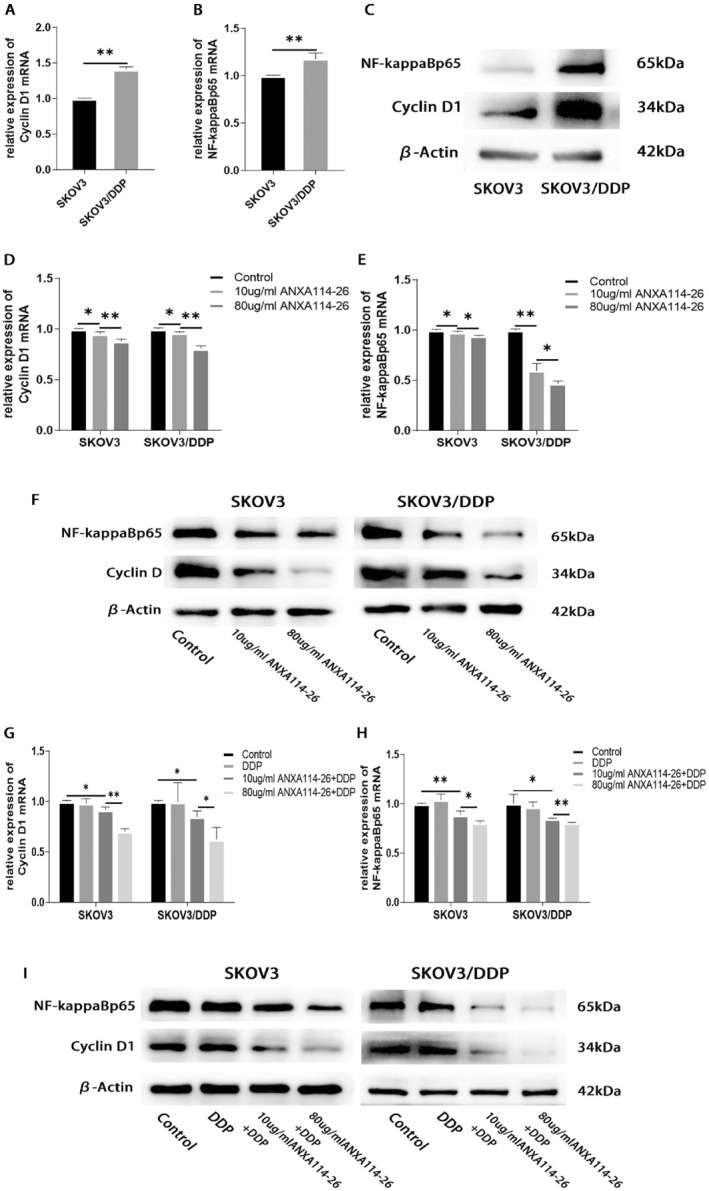
ANXA114‐26 may affect the proliferation and drug resistance of ovarian cancer cells through the Cyclin D1 and NF‐ĸBp65 signaling pathways. (A) Differences in mRNA expression of Cyclin D1 between SKOV3 and SKOV3/DDP cell lines. (B) Differences in mRNA expression of NF‐ĸBp65 between SKOV3 and SKOV3/DDP cell lines. (C) Differences in protein expression of Cyclin D1 and NF‐ĸBp65 between SKOV3 and SKOV3/DDP cell lines. (D) The relative expression of Cyclin D1 mRNA. (E) The relative expression of NF‐ĸBp65 mRNA. (F) Protein expression levels of Cyclin D1 and NF‐ĸBp65. (G) The relative expression of Cyclin D1 mRNA. (H) The relative expression of NF‐ĸBp65 mRNA. (I) Protein expression levels of Cyclin D1 and NF‐ĸBp65. The data were independently replicated in triplicate., *p < 0.05, **p < 0.01.

Drug resistance in tumor cells has been reported to be correlated with the expression levels of Cyclin D1 and NF‐ĸBp65.^22^, ^23^, ^24^, ^25^ Our results showed that the expression levels of Cyclin D1 and NF‐ĸBp65 in SKOV3/DDP cell line were higher than those in SKOV3 cell line (Figure 4A–C), suggesting that the elevated expression of Cyclin D1 and NF‐ĸBp65 may be related to drug resistance in ovarian cancer cells. After incubation with different concentrations of ANXA114‐26, qRT‐PCR and Western blot were used to detect the mRNA and protein expression changes of Cyclin D1 and NF‐ĸBp65. The results showed that the expression of Cyclin D1 and NF‐ĸBp65 decreased with the increase of ANXA114‐26 concentration (Figure 4D–F). We also examined the changes in the expression of Cyclin D1 and NF‐ĸBp65 in ovarian cancer cells treated with ANXA114‐26 in combination with cisplatin. The results showed that Cyclin D1 and NF‐ĸBp65 were no significant change in the cisplatin treatment group alone, compared with the control group, and the expression of Cyclin D1 and NF‐ĸBp65 decreased with the increase of ANXA114‐26 in the ANXA114‐26 combined with cisplatin treatment group (Figure 4G–I). The above results showed that cisplatin had no effect on the expression of Cyclin D1 and NF‐ĸBp65, while ANXA114‐26 combined with cisplatin could reduce the expression of Cyclin D1 and NF‐ĸBp65.

In summary, ANXA114‐26 may inhibit the proliferation of SKOV3 and SKOV3/DDP and reverse cell resistance by decreasing the expression of Cyclin D1 and NF‐ĸBp65.

### 3.6 ANXA114‐26 promoted apoptosis and reduced MRP1 expression in ovarian cancer cells by inhibiting the FPR/Cyclin D1/NF‐ĸBp65 pathway

**FIGURE 5:**
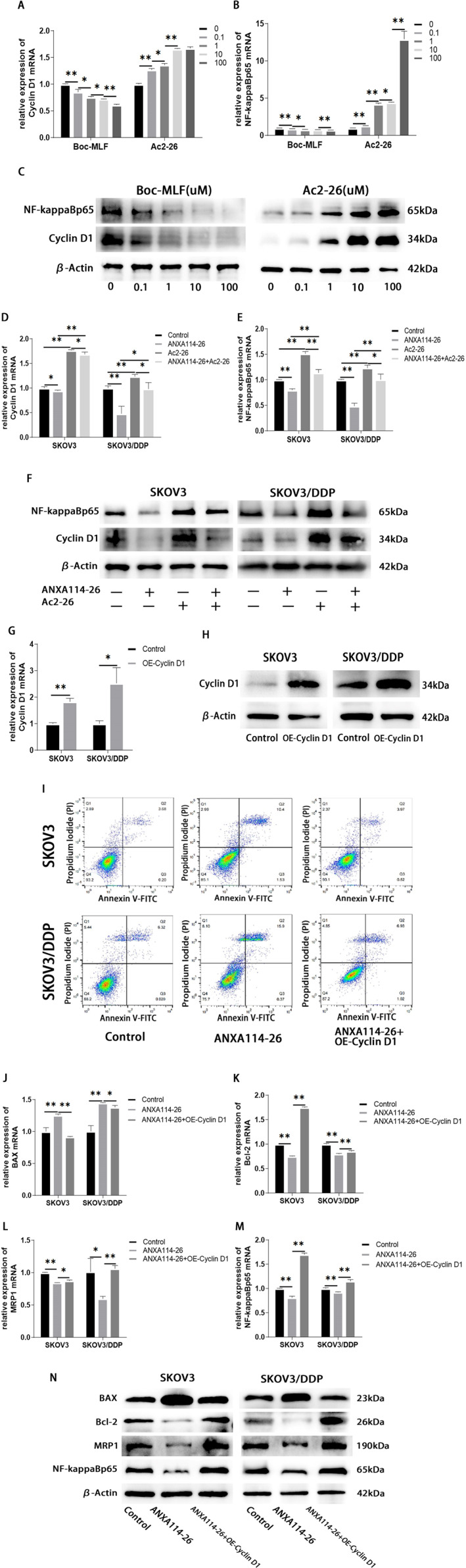
ANXA114‐26 promotes apoptosis and reduces MRP1 expression in ovarian cancer cells by inhibiting the FPR/Cyclin D1/NF‐ĸBp65 pathway. (A) The relative expression of Cyclin D1 mRNA. (B) The relative expression of NF‐ĸBp65 mRNA. (C) Protein expression levels of Cyclin D1 and NF‐ĸBp65. (D) The relative expression of Cyclin D1 mRNA. (E) The relative expression of NF‐ĸBp65 mRNA. (F) Protein expression levels of Cyclin D1 and NF‐ĸBp65. (G) Validation of mRNA expression of Cyclin D1 after plasmid transfection. (H) Validation of protein expression of Cyclin D1 after plasmid transfection. (I) The apoptosis of ovarian cancer cells was detected by Annexin V‐FITC/PI assay. (J) The relative expression of BAX mRNA. (K) The relative expression of Bcl‐2 mRNA. (L) The relative expression of MRP1 mRNA. (M) The relative expression of NF‐ĸBp65 mRNA. (N) Protein expression levels of BAX, Bcl‐2, MRP1 and NF‐ĸBp65. The data were independently replicated in triplicate., *p < 0.05, **p < 0.01.

We investigated the effects of Boc‐MLF and Ac2‐26 on the expression of Cyclin D1 and NF‐ĸBp65 in SKOV3/DDP cells. The results showed that the expression of Cyclin D1 and NF‐ĸBp65 in Boc‐MLF treated cells were reduced. The expression of Cyclin D1 and NF‐ĸBp65 in Ac2‐26 treated cells increased (Figure 5A–C). These results indicated that the inhibition of FPR1 by Boc‐MLF led to the decrease of Cyclin D1 and NF‐ĸBp65 expression, while the activation of FPR by Ac2‐26 promoted the expression of Cyclin D1 and NF‐ĸBp65 in ovarian cancer cells.

To investigate whether ANXA114‐26 regulates the expression of Cyclin D1 and NF‐ĸBp65 by competitively binding to FPR with Ac2‐26. We used qRT‐PCR and Western blot to detect whether ANXA114‐26 could reverse the promoting effect of Ac2‐26 on Cyclin D1 and NF‐ĸBp65, and the results showed that the expression levels of Cyclin D1 and NF‐ĸBp65 in the ANXA114‐26 + Ac2‐26 group were lower than those in the Ac2‐26 group and higher than those in the ANXA114‐26 group (Figure 5D–F). These results suggest that ANXA114‐26 reverses the promoting effect of Ac2‐26 on Cyclin D1 and NF‐ĸBp65 by competitively binding FPR to Ac2‐26.

In order to further clarify whether ANXA114‐26 inhibits cell proliferation and reduces MRP1 expression through CyclinD1, we detected the apoptosis and drug resistance changes of SKOV3 and SKOV2/DDP cells after overexpression of Cyclin D1 (Figure 5G–H). Flow cytometry results showed that compared with the ANXA114‐26 group, the apoptotic cells in the ANXA114‐26+OE‐Cyclin D1 group decreased, indicating that overexpression of Cyclin D1 could reverse the inhibitory effect of ANXA114‐26 on ovarian cancer cells (Figure 5I). The expression levels of BAX, Bcl‐2, MRP1 and NF‐ĸBp65 were detected by qRT‐PCR and Western blot, and the results showed that compared with the ANXA114‐26 group, the expression of BAX in the ANXA114‐26+OE‐Cyclin D1 group was decreased, and the expression levels of Bcl‐2, MRP1 and NF‐ĸBp65 were increased (Figure 5J–N). It indicated that ANXA114‐26 had multiple effect on the ovarian cancer cell proliferation, drug resistance, and downstream protein NF‐ĸBp65 through Cyclin D1.

## 4 DISCUSSION

A variety of mechanisms can contribute to chemotherapy resistance in patients with ovarian cancer. In addition to chemoresistance caused by intracellular changes in cancer, a growing body of studies has also shown that tumor microenvironment (TME) is associated with chemoresistance in cancer. For example, cytokines secreted by bone marrow stromal cells and fibroblasts can mediate drug resistance in tumor cells through corresponding receptors and cell signaling pathways^4^, ^5^, ^26^, ^27^;TME hypoxia can lead to increased drug resistance in tumor cells.^18^, ^28^ Similarly, tumor resistance can be mitigated by targeting changes in TME.^29^

In this study, we detected 41 differentially expressed peptides in the serum of chemotherapy‐sensitive/resistant patients with ovarian cancer. After searching relevant literature and databases, we preliminarily identified ANXA114‐26 as our research object. Subsequently, ANXA1 was found to be indeed associated with drug resistance in ovarian cancer.

Researchers have discovered some peptides that have the functions of promoting tumor cell apoptosis and inhibiting tumor cell proliferation, providing more options for tumor treatment^30^, ^31^。In this study, We found that ANXA114‐26 can inhibit ovarian cancer cell proliferation and reduce the expression of MRP1 alone or in combination with cisplatin, suggesting that ANXA114‐26 may be a potential treatment for ovarian cancer.

FPRs are a family of seven transmembrane G protein coupled receptors, consisting of three members: FPR1, FPR2, and FPR3 ^32^。ANXA1 is secreted outside the cell and exerts its biological function through the receptor FPR on the cell membrane.^8^, ^10^, ^14^, ^33^ Research has found that fMLF (a mature FPR1 activator) and ANXA1 derived from the supernatant of necrotic tumor cells can activate FPR1 in glioblastoma cell line U87, causing increased intracellular Ca2+mobilization and inducing tumor cell migration, invasion, proliferation, and colony formation ^10^。The experimental results of Babbin BA et al. showed that AnxA1, Ac2‐26, and other FPR agonists (fMLP and WKYMVm) activate FPR to induce intracellular calcium release in colorectal adenocarcinoma cell line SKCO‐15, leading to an increase in SKCO‐15 invasion ^33^。Studies have shown that the expression or functional status of FPR is related to drug resistance of tumor cells.^18^, ^34^ Our results showed that Boc‐MLF inhibited FPR1 and led to increased apoptosis and decreased MPR1 expression in SKOV3/DDP cells, while Ac2‐26 could activate FPR, resulting in decreased apoptosis and increased MRP1 expression. Simultaneous treatment of ovarian cancer cells with Ac2‐26 and ANXA114‐26 found that ANXA114‐26 reversed the Ac2‐26‐mediated increase in cell viability and drug resistance by competitively binding FPR to Ac2‐26. We hypothesized that ANXA114‐26 was able to bind FPR due to its homology to ANXA1, but the short amino acid sequence of ANXA114‐26 did not activate the downstream signaling pathway. When the concentration of ANXA114‐26 is higher, the binding rate to FPR is higher, which blocks the binding of ANXA1 secreted by tumor cells or TME‐related cells to FPR, so with the increase of the concentration of ANXA114‐26, the binding rate of ANXA1 to FPR decreases, and the apoptosis of ovarian cancer cells increases, and drug resistance decreases.

Factors influencing the tumor cell cycle are an important factor in altering drug resistance in tumor cells.^35^ As a component of the cell cycle mechanism, changes in the expression of cyclin D1 affect the cell cycle progression, thereby altering the killing effect of antitumor drugs on tumor cells.^24^, ^36^ Many studies suggest that the high expression of Cyclin D1 may be an important factor in promoting the resistance of tumor cells to chemotherapy drugs.^22^, ^23^, ^36^ In this study, we found that the expression of Cyclin D1 in SKOV3/DDP was higher than that of SKOV3; ANXA114‐26 decreases the expression of Cyclin D1 alone or in combination with cisplatin; ANXA114‐26 regulates the expression of Cyclin D1 through the FPR receptor. In addition, when Cyclin D1 was overexpressed, we found that the apoptosis rate of ovarian cancer cells was reduced and the expression of MRP1 was increased. Our results suggest that ANXA114‐26 reduces ovarian cancer cell proliferation and drug resistance by decreasing the expression of Cyclin D1.

NF‐ĸB is involved in many processes of tumor cytogenesis and development.^37^, ^38^ Studies have shown that reduced activity of NF‐ĸB can inhibit the growth of triple‐negative breast cancer cells^39^; high expression of NF‐ĸB inhibits apoptosis in lung cancer cells and thyroid cancer cells.^40^ In conclusion, a large number of studies have found that the activation or overexpression of the NF‐ĸB signaling pathway promotes tumor progression. In this experiment, we found that ANXA114‐26 inhibits the expression of NF‐ĸBp65 by FPR, and the expression of NF‐ĸBp65 increased when Cyclin D1 was overexpressed. Our study demonstrated that NF‐ĸBp65 was involved in the regulation of ANXA114‐26 on ovarian cancer cell proliferation and drug resistance. However, the specific mechanism of NF‐ĸB in the regulation of ANXA114‐26 on ovarian cancer cells will be further explored in future experiments.

In summary, we discovered a small molecule peptide called ANXA114‐26, which is an N‐terminal peptide derived from ANXA1. ANXA114‐26 can competitively bind to the cell membrane receptor FPR with ANXA1 in the TME. ANXA114‐26 promotes apoptosis and reduces drug resistance in ovarian cancer cells through the FPR/Cyclin D1/NF‐ĸBp65 pathway. ANXA114‐26 plays a role in the extracellular location. Further study is needed to find the optimal concentration of ANXA114‐26 for reversing chemo‐resistance of ovarian cancer and inhibiting the growth of ovarian cancer cells.

## AUTHOR CONTRIBUTIONS

Conceptualization, Nana Li and Peihua Yan; Writing – original draft, Nana Li and Peihua Yan; Data curation, Ling Guo; Funding acquisition, Yajuan Su; Investigation, Nana Li; Project administration, Huiyan Wang; Resources, Baohong Cui; Supervision, Yajuan Su and Lichen Teng.

## CONFLICT OF INTEREST STATEMENT

The authors declare that they have no conflict of interests.

## ETHICS STATEMENT

All procedures performed in studies involving human participants were in accordance with the ethical standards of the institutional and/or national research committee and with the 1964 Helsinki declaration and its later amendments or comparable ethical standards.

## CONSENT FOR PUBLICATION

All authors consent for publication.

## Data Availability

The data supporting this study are all included in this article.
